# The Responsibility of Silence

**Published:** 1909-10-16

**Authors:** 


### The Hospital

A Journal of

The Medical Sciences and Hospital Administration.

New Series. No. 138, Vol. VI. [No. 1210, Vol. XLVII.] SATURDAY, OCTOBER 16, 1909.

### THE RESPONSIBILITY OF SILENCE.

Great responsibilities in recent years have devolved upon the honorary and resident staff of our hospitals, both collectively and as individuals. These responsibilities have quickened the intelligence, raised the standard and character of much of the work, and in the best administered hospitals have brought with them a spirit which has changed these institutions from places which repelled rather than attracted, to centres of life in the highest and best meaning of the term.

The revolution which has been effected, by increasing the responsibility of individuals, makes it well to consider whether or not silence on their part may ever be carried dangerously near to the point where it becomes a dereliction of plain duty. The responsibility of each member of the honorary and resident medical staff at the present time is not free from difficulty, and calls for splendid discipline, but discipline as represented by silence may be carried a great deal too far. Discretion is a great virtue which too few possess in these days, and abstinence from speech may often be both prudent and wise. There is a silence, however, resting upon a false feeling of *esprit* which may prove harmful to the individual and fatal to the exercise of the highest duty. All good men and women are capable of knowing and observing the distinction of right from wrong in conduct. No men or women are true to their highest instincts, or fulfil their highest duty, when they fail to recognise their obligation to break silence when they see practices continuing, or acts committed, which are morally wrong. A direct responsibility attaches to the splendid confidence which is reposed by the managers of our hospitals, by the patients and their friends, and by the public, in each individual member of the honorary and resident medical staff as members of a great and honourable profession. We would venture in this connection to ask all practitioners to take counsel with themselves, to consider this matter in all its bearings, to look back on the whole of their experience in hospital work, and to satisfy themselves whether or not they have ever carried the responsibility of silence too far.

Our thoughts have been turned in this direction owing to matters which have arisen in the course of our work. Let us indicate a few of the difficulties by quoting cases which may occur in the ordinary daily life of any honorary or resident medical officer of a hospital. They are merely suggestive; they only indicate points we have in view and are by no means exhaustive, but they will suffice. The development of the work may move the management of a hospital to make arrangements for the establishment of an ophthalmic department. It may happen, as has often occurred, that there is a difficulty about providing an ophthalmic ward, and an ophthalmic surgeon may be offered some beds in one of the general wards to which septic cases may be admitted. Is an ophthalmic surgeon justified in carrying out the more serious ophthalmic operations and placing his patients after operation in any such beds? If he declines, he entails the risk of being deprived of beds altogether and so unable properly to discharge his duties to the hospital patients, thus being placed in a great difficulty involving considerable responsibility. Our answer, and we believe it is an answer which will be supported by the best ophthalmic surgeons, is that a member of the profession, circumstanced as we have described, cannot accept the responsibility of silence, but must refuse to submit his patients to the risks involved by treating them under the conditions named.

Again, it may happen that a member of the staff may become gradually inefficient or incapable, rendering it essential, if the best interests of the hospital patients are to be the first consideration, that steps shall be taken to discontinue his attendance and treatment. Are his colleagues, and are the resident medical staff, justified in remaining silent and sanctioning by their presence treatment which they believe wrong or dangerous or undesirable in a patient's interest? Once again we say the responsibility of silence is one which no high-spirited, manly gentleman must permit himself to take.

Again, members of the medical staff may be conscious that one or more of its number, though otherwise highly educated and capable men, do not possess the ability to perform operations with sufficient despatch, and in practice keep the patient under an anæsthetic for a dangerous, if not fatal, period. Are there any ethical or other reasons which justify the members of the medical staff or the medical board in allowing such things to continue indefinitely at their hospital by embracing the responsibility of silence? We venture to say, in view of the enormous growth in the number of operations and in the readiness with which they are now performed, that the members of a medical staff who knowingly remain silent in such circumstances fail clearly in their duty. Indeed it is difficult to exaggerate the contempt which every individual amongst them must feel for himself should he in any of the circumstances named fail to recognise his responsibility. His own conduct as an individual makes it imperative that he should not remain silent. The responsibility of silence, so far as the members of the staff of our hospitals are concerned, is indeed great, and we have every confidence that the views we have ventured to express represent the best opinion of the profession in this country to-day.

